# Feasibility and Reliability of Physical Fitness Tests among Colombian Preschool Children

**DOI:** 10.3390/ijerph16173069

**Published:** 2019-08-23

**Authors:** Julio Cesar Amado-Pacheco, Daniel Humberto Prieto-Benavides, Jorge Enrique Correa-Bautista, Antonio García-Hermoso, César Agostinis-Sobrinho, Alicia María Alonso-Martínez, Mikel Izquierdo, Robinson Ramírez-Vélez

**Affiliations:** 1 Center of Studies in Physical Activity Measurements (CEMA), School of Medicine and Health Sciences, University of Rosario, 111221 Bogotá, Colombia; 2 Department of Health Sciences, Public University of Navarra, 31006 Pamplona, Navarra, Spain; 3 Laboratorio de Ciencias de la Actividad Física, el Deporte y la Salud, Facultad de Ciencias Médicas, Universidad de Santiago de Chile, USACH, 9160030 Santiago, Chile; 4 Faculty of Health Sciences, Klaipeda University, 92294 Klaipeda, Lithuania; 5 CIBER of Frailty and Healthy Aging (CIBERFES), Instituto de Salud Carlos III, 28001 Madrid, Spain

**Keywords:** reliability, health-related physical fitness, morphological, preschooler

## Abstract

The aim of the study was to assess the feasibility and reliability of physical fitness field tests used in the “Fuprecol kids” study among Colombian preschool children aged 3–5 years. A total of 90 preschoolers aged 3–5 years participated in the study. Weight, height, waist circumference, cardiorespiratory fitness (CRF), musculoskeletal fitness (handgrip strength and standing broad jump), speed–agility (4 × 10 m shuttle run), and flexibility (sit and reach test) components were tested twice (two weeks apart). The feasibility of the tests (preschoolers able to complete the test) ranged from 96% in the CRF test to 100% in the musculoskeletal fitness, speed–agility, and flexibility tests. Overall, the %TEMs were 0.625% for the weight, 0.378% for the height, 1.035% for the body mass index, and 0.547% for the waist circumference. In addition, all tests were substantial reliable, for CRF (in stages and laps, concordance correlation coefficient = 0.944 and 0.941, respectively) in both sexes and flexibility (concordance correlation coefficient = 0.949) in girls. There were no significant differences in fitness test–retest mean differences in the boys (*p* > 0.05), except in CRF (laps *p* = 0.017). In girls, there were differences in CRF (stages (*p* = 0.017) and laps (*p* = 0.013)), and flexibility (*p* = 0.002) variables. The results from this study indicate that the “Fuprecol kids” battery of tests, administered by physical education teachers, was reliable and feasible for measuring components of physical fitness in preschoolers in a school setting in Colombia.

## 1. Introduction

Motor fitness (i.e., speed–agility), musculoskeletal fitness (MSF), and CRF are the powerful health-related fitness components in youths [1]. In particular, low CRF and low MSF are independently associated with increased cardio-metabolic risk [2,3,4,5] and mortality [6,7] in both youth and adult populations. It has been consistently reported that a higher adiposity (i.e., central abdominal or fat mass) and metabolic risk factors are associated with lower CRF and MSF levels in young people [8]. Based on this evidence, youth fitness assessment guidelines have called for a better understanding of the inter-relationship between physical fitness and body composition [9].

Owing to the importance of physical fitness components for current and future health in youths, it is important that intervention studies use feasible, reliable, and valid measures to assess fitness. Consequently, the assessment of physical fitness in this population is important for understanding the relationship between interventions. A review of previous literature relevant to establishing a fitness test battery in early ages revealed the only reference used for the field-based fitness-test battery in preschool children (<6 years old) was the PREFIT (preschool children fitness testing) battery [10]. 

Most studies on this topic to date are published on European preschoolers and therefore other studies are needed to confirm the findings in their Latino counterparts. This is important to assess, particularly in the context of a low-to-middle income country setting like Colombia, where most of the burden occurs due inactivity and related non-communicable chronic diseases. While information in preschools is sparse, a recent longitudinal study published by Henriksson et al. [11] confirmed the importance of physical fitness early in life, suggesting that better physical fitness at 4.5 years of age is associated with lower fat mass and higher fat-free mass one year later. The physical fitness tests, included in the “Fuprecol study” [12], have been previously validated in children and adolescents. The results from this study indicate that the “Fuprecol study” health-related physical fitness battery was reliable for measuring health-related components of fitness in children and adolescents aged 9–17.9 years old in a school setting in Colombia [12]. However, the feasibility and reliability of physical fitness tests has not been explored in Colombian preschool children. 

Based on current evidence regarding fitness and fatness as potentially modifiable risk factors related to health and disease in preschoolers [13], the assessment of physical fitness from an early age is relevant from a public health point of view. 

In Colombia, a region which has undergone a well-documented epidemiologic transition fueling a non-communicable diseases epidemic, relatively little research on physical activity and physical fitness exists [14,15]. This is important to assess, particularly in the context of a low-to-middle income country setting like Colombia [12]. Both feasibility and reliability are characteristics that need to be assured for any measurement tool [9]. This study would be of great importance for use in future studies in the Latin-American context and further clarifies the role of physical fitness in preschoolers for later health. Therefore, the aim of the study was to assess the feasibility and reliability of physical fitness field tests used in the “Fuprecol kids” battery among Colombian preschoolers.

## 2. Materials and Methods

### 2.1. Design Study and Subjects

The present study was performed under the framework of the “Fuprecol kids” study. That study used data from a convenient sample of preschoolers in the third to fifth year of elementary school (90 healthy preschool children; 48 boys and 42 girls), who were enrolled in public schools in the city of Bogota, Colombia, (hereinafter called preschoolers, between 3–5 years old). The recruitment period lasted from June 2017 to January 2018. This study was approved by the Research Ethics Committee of the University of Rosario (Code Nº CEI-UR DVO005-1-269-CEI875, date 09/02/2018) in accordance with the latest revision of the Declaration of Helsinki. All participants and their parents/legal guardians provided written informed consent before entering the study. 

### 2.2. Procedures

Consistent with a previous systematic review [16] and recommendations [9], we restricted our analysis to field-based tests that have demonstrated adequate levels of criterion-related validity [10,17,18,19] and reliability [12] in the assessment of four components of the “Fuprecol kids” battery of tests. Thus, the following tests were included: Weight, height, and waist circumference (WC) to assess the morphological component; the PREFIT 20 m shuttle run test (PREFIT 20m-SRT) to assess the CRF component; standing long jump and handgrip strength tests to assess the musculoskeletal component (upper and lower limbs, respectively); 4 × 10 m shuttle run test (4 × 10 m SRT) to assess the speed–agility component; and finally, the sit and reach test to assess the flexibility component.

At each school, a team of trained CEMA (in Spanish, Centro de Estudios en Medición de la Actividad Física: https://www.urosario.edu.co/CEMA/Inicio/) center evaluators administered the tests in partnership with the physical education instructor. Data were collected by a blinded trained staff member according to standardized protocol [10,12] prior to baseline and two weeks later. To determine the test–retest reliability of the “Fuprecol kids” battery of tests, the assessments were administered twice (two weeks apart as previously done in similar reliability studies [12] under the same physical conditions and by the same physical education instructor. Re-testing was performed at the same time of day to minimize circadian rhythm variability. To assess the feasibility of the “Fuprecol kids”, we measured major problems detected in each physical fitness test.

The sample size was determined as by Walter et al. [20], with two replicates per subject; the expected reliability coefficient had to be at least 0.80 to 0.90 (H1: ρ1 = 0.8–0.9) or higher to be minimally acceptable, α = 0.05 and β = 0.2 (corresponds to 80% power); this would require a total of 80 subjects. Using a 10% over-estimate to account for poor response, the final target sample size was 90.

Morphological component: The measurement sessions took place within the normal routine of the schooldays by research assistants. They were asked to take the measurements with a calibrated flat beam scale for mobile use (Tanita ® SC-331S, scale division: 100 g, capacity: 200 kg), a stadiometer (SECA 217, graduation length: 1 cm, range: 20–205 cm), and measuring tape (Lufkin W606PM®, Parsippany, New Jersey, USA, graduation length: 0.1 cm, range: 1–200 cm). Height was measured to the nearest 0.1 cm using a stadiometer, without shoes and with light clothing. WC was measured using a metal tape measure at the level of the umbilicus zone in the horizontal plane. Body mass index, BMI (kg/m^2^) was calculated as weight divided by height squared.

Physical fitness component: Details of the collection physical fitness methods have been published previously by Cadenas-Sanchez et al. [9] in The PREFIT Project framework (http://profith.ugr.es/prefit), Supplementary File S1. Briefly, the CRF was estimated from the number of laps and stages obtained in the PREFIT 20m-SRT [10]. Lower limbs were estimated from standing broad jump (cm). The result was recorded in centimeters and analyzed according to a previous publication [10]. The handgrip test (kg) was employed to evaluate upper limbs. The average of the values for the left and right sides was taken as the final value for handgrip strength to avoid differences between sides in kilogram (kg), without consideration for hand dominance. The 4 × 10 m SRT was employed to evaluate speed of movement, agility, and coordination assessment [10,11,12]. The best of two attempts was recorded (seconds). Flexibility was measured by the sit-and-reach test, using the procedures outlined in the Australian Council for Health, Physical Education and Recreation battery [21]. The result was recorded directly from the meter on the device [12]. The preschoolers wore sports clothing and footwear during testing.

### 2.3. Statistical Analyses

The data are presented as the means ± SD, unless otherwise stated, for the whole sample and stratified by sex. The agreement between test–retest trials of all tests (CRF, musculoskeletal, speed–agility, and flexibility components) was assessed following the Bland–Altman method [22]. The analysis measures bias as estimated from mean differences, the 95% confidence interval for bias, the limits of agreement, and ±1.96 SD of the difference. Cohen´s d was computed to quantify the magnitude of the difference between test and retest. Sex differences of the studied health-related physical fitness tests were analyzed by a t-test on inter-trial difference (test 2−test 1, hereafter called T2−T1). 

The technical error of measurement (TEM), which is an accuracy index, was also calculated in the morphological component and fitness components such as musculoskeletal (handgrip strength and standing long jump) and motor/flexibility (speed–agility: 40 m SRT and sit and reach test). The formula for TEM calculation is ∑D^2^/2N. The lower the TEM obtained, the better the reliability. The acceptable ranges for relative TEM using beginner anthropometrist levels for intra-test (T2−T1) is <1.5%. The absolute TEM was converted into relative TEM (%TEM) using the equation: %TEM = (TEM/mean) × 100. Relative reliability (R) of the components of the fitness tests such as CRF, musculoskeletal fitness, speed–agility, and flexibility were determined by the intra-class correlation coefficient (ICC).

To determine the concordance correlation between test–retest measures, we used Lin’s concordance correlation coefficient (pc). The feasibility of each test was calculated as the percentage of participants who were able to complete the tests satisfactorily on test T1 and retest T2 occasions. All analyses were performed with statistical programs MedCalc 16.8.4® (Ostend, Belgium) and IBM SPSS Statistics 24 software for Windows (SPSS, Chicago, Illinois, USA). For all analyses, the significance level was 0.05.

## 3. Results

The characteristics for the four components of the “Fuprecol kids” study (mean value ± SD) were assessed twice, as well as the mean inter-trial difference in the study, and are shown in Table 1. There were significant differences in the physical fitness component between the first trial and the second trial in terms of anthropometric characteristics (height and WC, *p* < 0.004) and cardiorespiratory component (stage and laps, *p* < 0.002). 

Regarding feasibility, physical fitness testing in preschoolers using the “Fuprecol kids” battery was feasible, without any major problems detected when it was implemented in the CRF, musculoskeletal fitness, speed–agility, and flexibility tests (96 to 100%). The lowest feasibility was observed in the CRF test (96%). The only problem for those children who did not complete the CRF test was discomfort in breathing, (*n* = 3 participants).

Table 2 shows the reliability statistics by sex. Overall, there were no significant differences in test–retest mean differences in the boys (*p* > 0.05), except in WC (*p* = 0.001) and CRF (laps *p* = 0.017). In girls, there were differences in WC (*p* = 0.001), CRF (stages (*p* = 0.017) and laps (*p* = 0.013)), and flexibility (*p* = 0.002) variables. 

Table 3 shows the inter-observer TEM, %TEM and intra-class coefficient for each physical fitness component. The relative TEMs were 0.625% for the weight, 0.378% for the height, 1.035% for the body mass index, and 0.547% for the WC. The lower the %TEM obtained, the better the reliability (the acceptable range for inter-trial tests is <1.5%). In addition, we found that all the relative reliability values (for intra-observer) were above the ICC 0.97 suggested cut-off in fitness components such as CRF and musculoskeletal fitness.

**Figure 1:**
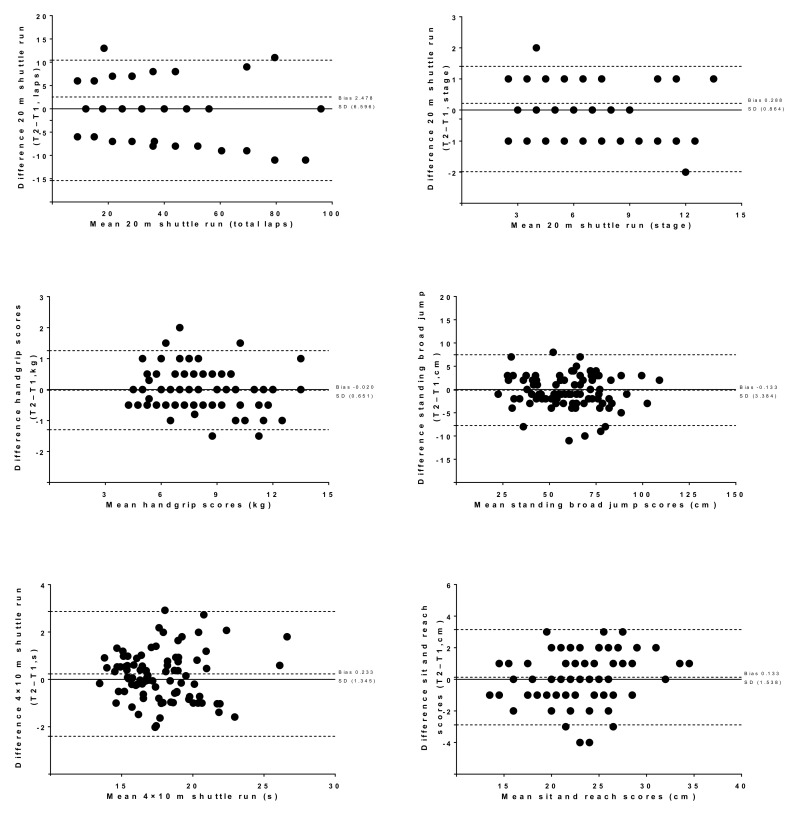
Bland–Altman plot of the “Fuprecol kids” battery (preschool children fitness testing) 20 m shuttle run test (laps and stage), handgrip strength, standing broad jump, 4 × 10 m shuttle run test, and sit and reach test among Colombian preschool children: The “Fuprecol kids” study. The central dotted line represents the mean differences between the second trial (T2) and the first trial (T1); the upper and lower dotted lines represent the upper and lower 95% limits of agreement (mean differences ± 1.96 SD of the differences), respectively.

The Bland–Altman plots (Figure 1) graphically show the reliability patterns, in terms of systematic errors (bias or mean inter-trial differences) and random error (95% limits of agreement), of the “Fuprecol kids” battery of tests. The systematic error when fitness assessment was performed twice was nearly zero for all the tests (range −0.133 and 0.233).

## 4. Discussion

The main finding of our study shows that the “Fuprecol kids” battery of tests administered by physical education teachers is reliable for assessing the levels of physical fitness in preschoolers in a school environment in the Colombian setting. The TEM, %TEM, ICC, and pc values for all the physical fitness tests in the present study were substantial and represented a good reliability. Despite the significant differences (i.e., height, WC or CRF) in clinical terms, these tests showed a low mean difference between the test and retest for the whole sample.

A test is considered to be reliable when an individual obtains similar results when performing the test on two or more occasions under the same conditions and in close succession. Concerning reliability, we observed a systematic error of 2.50 (*p* = 0.017) and 2.45 (*p* = 0.013) laps in boys and girls in the CRF test, respectively. These findings concur with those reported by Cadenas-Sanchez et al. [10] in Spanish preschoolers. This study reported a mean difference of two laps in the CRF test, considering the different age groups of the participants and that assessments were two weeks apart. 

For the MSF components, we found adequate reliability patterns, in terms of systematic errors (bias) and random error (95% limits of agreement). Scientific evidence indicates that strength tests have produced moderate test–retest reliability. The handgrip strength test reported a mean difference of −0.02 (0.65) kg and a substantial correlation (full sample pc = 0.970 and 0.930 for boys and girls, respectively). The mean difference and concordance correlation coefficient were better than those showed by Spanish preschoolers [10], 0.38 kg and 0.05 kg for boys and girls (pc = 0.859), respectively, but were similar to those that were reported among Colombian children and adolescents [12]. Therefore, these results confirm that, when performing the handgrip strength test with the TKK dynamometer adapted to the hand size, the agreement between test and retest is the same throughout the range of measured values [16,23]. Another possible reason for the divergence between studies might be methodological differences (for example, variability in the equipment used and the protocol for measuring handgrip strength or grip span). In addition, evidence has shown no significant differences in test–retest for the standing broad jump for European [16,23] and Colombian [12] youths; this is in agreement with our results in preschool children. However, for this population, other studies show contradictory results. For example, Oja and Jürimäe [19] showed that the standing broad jump was highly reliable in four- and five-year-old preschool children, but the coefficient of variation was higher in girls than in boys. In contrast, for Spanish preschoolers, the authors reported a systematic error of 7.31 cm, suggesting that the reliability of this test is questionable, due to the higher coordination patterns needed for the standing long jump test and the difficulty observed in the preschool stage of performing it correctly [12]. 

Different fitness components such as flexibility, muscular fitness, and speed–agility mean values have been observed in different countries, as reported previously, [8,13,15,20] but the nature of these differences is not known. Similar findings were reported by Castro-Piñero et al. [24], who observed sex differences during the stage from childhood to adolescence. For example, differences in grip strength or CRF values between Colombian preschool children, who are from a less-developed country (this study), and individuals in developed countries (i.e., European children) may be due to a number of factors, although it is uncertain which of the three factors, genetic, environmental, or biological, are more decisive for fitness results [25]. Moreover, beyond ethnic differences in height and in health status and function, there are well-recognized differences in dietary intake between different countries, and this variation might also explain differences in muscular fitness [26]. CRF has also been related to nutrition status and is reported to have a positive influence on other fitness components. 

Likewise, for the 4 × 10 m-SRT, the mean difference between measurements in the 4 × 10 m SRT was 0.23 (1.34) s, with a lower concordance correlation coefficient (pc = 0.890). Other authors reported the reliability of this test in preschool years and concluded that the test showed an acceptable reliability [10,19]; therefore, this test seems to be considered easy to measure.

Lastly, the reliability of the sit and reach test was analyzed in previous studies [12,19]. Regarding feasibility, Cornbleet and Woolsey [26] observed a significant correlation between the passive straight leg raise and the sit-and-reach test in children (r = 0.76), and suggested that both the forward reach score and pelvic angle reflect the hamstring muscle extensibility. Contrary to our findings, Oja and Jürimäe [19] demonstrated good test–retest reliability in 61 boys and girls aged 4–5 years (r = 0.75 to 0.93). The present study shows a systematic error of 0.59 (1.19) cm in girls (*p* = 0.002), reaffirming the results for older Colombian schoolchildren [12]. Since the hamstring extensibility and spinal posture are different among different age groups, additional studies are needed for preschoolers [25]. Finally, the comparison of the reliability of fitness tests between the “Fuprecol kids” study and the PREFIT study are shown in Supplementary File S2.

The present study showed that the intra-rater and inter-rater TEM and R% values were above the required levels. An allowance for measurement error might be up to 10% of the observed variance, which is equivalent to an R value of 90% or greater [27]. Specifically, the lower the %TEM obtained, the better the reliability (the acceptable range for inter-trial tests is <1.5%) [28]. Our results are similar to those found in other studies carried out with Colombian children and adolescents [12] and European adolescents [29,30,31,32]. In Colombian youths, older than those in the present study, Ramirez-Vélez et al. [12] reported that TEMs were small and reliability was greater than 95% in all cases for height and the waist and hip circumferences. Another study in preschoolers also showed similar reliability for anthropometric measures in both sexes [10].

Our study has several limitations. First, the study was not planned to be representative with respect to the broad range of variables that we investigated, but, given the "feasibility and reliability approach", this limitation not compromise the results obtained when validating our results. This was not feasible within our “Fuprecol kids” battery sample but may be investigated in later follow-ups of the cohort. Second is the lack of nationally representative samples. Thus, it might be questioned whether the present findings truly characterize the entire population of children living in Bogotá, Colombia. Third, the difficulty in differentiating between motivation and performance limitations is another study limitation to acknowledge [33]. Another limitation was not considering the cognitive performance for evaluating and understanding the instructions of the fitness test for the purposes of this study. Notwithstanding such limits, the results of this study seem to be in line with other studies in the literature [2,10,12,19]. The main strengths of this study are the standardized use of well-known and validated health-related fitness tests and a strong statistical method to obtain feasibility and reliability in the fitness tests.

## 5. Conclusions

In summary, the “Fuprecol kids” battery of tests is feasible and reliable for assessing the levels of physical fitness in preschoolers in a school environment in the Colombian setting. Despite the CRF test showing differences in test–retest mean differences in our study, in clinical terms these differences might not be meaningful. Additionally, these data of children aged 3–5 years complement the study published by Cadenas-Sanchez et al. [9] in The PREFIT Project framework (http://profith.ugr.es/prefit) from 10 different cities/towns in Spain. Thus, the results of this study contribute to the current body of literature by presenting a "feasibility and reliability approach" for Colombian preschoolers.

## Figures and Tables

**Table 1:** Test (T1), retest (T2), mean differences (T2–T1), and concordance correlation coefficient (pc) of “Fuprecol Kids” battery.

Component	1st Trial (T1)	2nd Trial (T2)	Mean Differences (Standard Deviation T2–T1) *	*p* Value	Effect Size	pc
**Morphologic**						
Age (years)	4.00 (0.82)	-	-	-	-	-
Weight (kg)	17.91 (2.82)	17.92 (2.81)	0.00 (0.14)	0.484	0.074	0.998
Height (m)	1.05 (0.07)	1.05 (0.07)	−0.00 (0.01)	0.004	0.314	0.997
Body mass index (kg/m^2^)	16.01 (1.53)	16.05 (1.52)	0.04 (0.21)	0.073	0.191	0.990
Waist circumference (cm)	52.50 (4.78)	52.27 (4.80)	−0.22 (0.29)	0.001	0.764	0.997
**Cardiorespiratory**						
20 m shuttle run (laps)	32.61 (19.98)	35.08 (21.99)	2.47 (6.59)	0.001	0.311	0.944
20 m shuttle run (stage)	5.88 (2.58)	6.17 (2.73)	0.28 (0.86)	0.002	0.334	0.941
**Musculoskeletal**						
Handgrip (kg)	7.77 (2.27)	7.75 (2.21)	−0.02 (0.65)	0.771	0.056	0.957
Standing broad jump (cm)	60.90 (18.71)	60.76 (18.73)	−0.13 (3.88)	0.745	0.034	0.978
**Motor and Flexibility**						
4 × 10 m shuttle run (s)	17.82 (2.69)	18.05 (3.11)	0.23 (1.34)	0.102	0.174	0.890
Sit and reach (cm)	23.32 (3.93)	23.45 (4.25)	0.13 (1.53)	0.412	0.087	0.929

Data in mean (SD). * Dependent t-tests were used to evaluate mean differences in the average T2 and T1 values.

**Table 2:** Test (T1), retest (T2), mean differences (T2–T1), and concordance correlation coefficient (pc) of “Fuprecol kids” battery, by sex.

Component	1st Trial (T1)		2nd Trial (T2)		Mean Differences (Standard Deviation T2–T1) *			
	Boys (*n* = 48)	Girls (*n* = 42)	Boys (*n* = 48)	Girls (*n* = 42)	Boys	*p* Value, (pc), Effect Size Boys	Girls	*p* Value, (*pc*), *Effect Size* Girls
**Morphologic**								
Age (years)	4.04 (0.82)	3.95 (0.82)	-	-	-	-	-	-
Weight (kg)	18.56 (2.93)	17.35 (2.73)	18.54 (2.91)	17.35 (2.69)	−0.01 (0.15)	0.422 (0.998) 0.117	−0.01 (0.13)	0.909 (0.998) 0.018
Height (m)	1.07 (0.07)	1.04 (0.06)	1.06 (0.07)	1.04 (0.06)	−0.01 (0.01)	0.031 (0.997) 0.321	−0.01 (0.04)	0.056 (0.997) 0.303
Body mass index (kg/m^2^)	16.13 (1.33)	15.87 (1.75)	16.16 (1.30)	15.92 (1.74)	0.03 (0.23)	0.307 (0.983) 0.149	0.04 (0.18)	0.105 (0.994) 0.256
Waist circumference (cm)	52.61 (4.93)	52.37 (4.67)	52.35 (4.97)	52.18 (4.65)	−0.25 (0.23)	0.001 (0.997) 1.050	0.30 (0.98)	0.001 (0.996) 0.562
**Cardiorespiratory fitness**								
20–m shuttle run (stage)	6.56 (2.87)	5.11 (1.95)	6.81 (3.12)	5.45 (2.00)	0.25 (0.86)	0.055 (0.955) 0.289	0.33 (0.87)	0.017 (0.889) 0.379
20–m shuttle run (laps)	37.91 (22.71)	26.54 (14.34)	40.4 (25.73)	29.00 (14.85)	2.50 (7.00)	0.017 (0.953) 0.291	2.45 (6.18)	0.013 (0.897) 0.381
**Musculoskeletal**								
Handgrip strength (kg)	8.19 (2.46)	7.30 (1.95)	8.10 (2.39)	7.35 (1.92)	−0.08 (0.58)	0.303 (0.970) 0.222	0.05 (0.72)	0.610 (0.930) 0.102
Standing broad jump (cm)	64.22 (17.63)	57.09 (19.39)	63.66 (17.75)	57.45 (19.47)	−0.56 (3.48)	0.268 (0.980) 0.162	0.35 (4.28)	0.592 (0.975) 0.083
**Motor and Flexibility**								
4 × 10 m shuttle run (s)	17.27 (2.58)	18.44 (2.71)	17.34 (2.44)	18.87 (3.58)	0.06 (0.96)	0.650 (0.926) 0.066	0.42 (1.67)	0.103 (0.854) 0.257
Sit and reach (cm)	23.45 (3.83)	23.16 (4.07)	23.18 (4.26)	23.76 (4.27)	−0.27 (1.69)	0.274 (0.910) 0.160	0.59 (1.19)	0.002 (0.949) 0.501

Data in mean (SD). * Dependent t-tests were used to evaluate mean differences in the average T2 and T1 values.

**Table 3:** Intra-observer TEM, relative TEM (%), and intra-class coefficient for fitness component assessments of “Fuprecol kids” battery.

Components	Mean (SD)	Intra-Qbserver (T2 vs. T1)	
		Absolute TEM	Relative TEM
**Morphologic**			
Weight (kg)	17.99 (2.87)	0.112	0.625
Height (m)	1.06 (0.07)	0.004	0.378
Body mass index (kg/m^2^)	16.03 (1.53)	0.166	1.035
Waist circumference (cm)	52.39 (4.80)	0.287	0.547
		**Absolute TEM**	**ICC**
**Cardiorespiratory fitness**			
20 m shuttle run (stage)	5.89 (2.58)	−	0.973
20 m shuttle run (laps)	33.85 (20.99)	−	0.975
**Musculoskeletal**			
Handgrip strength (kg)	7.77 (2.23)	0.502	0.984
Standing broad jump (cm)	58.75 (18.53)	4.978	0.989
**Motor and Flexibility**			
4 × 10 m shuttle run (s)	17.94 (2.90)	1.052	0.951
Sit and reach (cm)	22.69 (4.14)	1.687	0.964

TEM: Technical error of measurement. (−) It is based on at least two measurements taken of the same child by the same observer (intra-observer variability), except the CRF, which was only taken once.

## Supplementary Materials

The following are available online at https://www.mdpi.com/1660-4601/16/17/3069/s1, Supplementary Files S1 and S2.
