# Supplementary material for: Feasibility and Reliability of Physical Fitness Tests among Colombian Preschool Children
Source: Int J Environ Res Public Health. 2019 Aug 23;16(17):3069. doi: 10.3390/ijerph16173069 (PMC6747194; doi:10.3390/ijerph16173069)
Supplement: Supplementary file 1 [file ijerph-16-03069-s001.pdf]

Additional file 1

Aerobic fitness component

Cardiorespiratory fitness was assessed with *PREFIT 20 m shuttle run* (1). The testing was carried on inside a school in open field. In short, preschoolers ran in a straight line between two lines 20 m apart while keeping pace with pre-recorded audio signals. All children were asked to run back and forth between two parallel lines, 15 m or 20 m apart, following the pace of an audio signal that began at a speed of 6.5 km/h and increased by 0.5 km/h at 1-minute intervals. Two evaluators ran together with a small group of children, in order to provide an adequate pace. The number of laps (shuttles) and stage completed was recorded for each participant and participants were encouraged at all times to produce a maximal effort. The audio signal is freely available in both languages, Spanish and English for download at the website of Universidad de Granada research group (http://profith.ugr.es/recursos-prefit?lang=en).

Musculoskeletal component

Lower limbs, standing broad jump (cm). The participant stood behind the starting line and was instructed to push off vigorously and jump as far as possible. Before performing this test, a familiarization time (with one attempt) was applied. Preschoolers flexed their knees, held their arms forward and parallel to the ground, and jumped without momentum. The test was performed twice, and the best attempt was recorded. The distance was measured by the appraiser (who was positioned next to the participant) and was measured from the scribe line to the point where the back of the heel touched the ground (2). The result was recorded in centimeters and analyzed according to a previous publication (1).

Upper limbs, handgrip strength (kg). Grip strength was assessed by the handgrip test using a hand dynamometer with an adjustable grip T-18 TKK Analog Grip Tester SMEDLY III® (Takei Scientific Instruments Co., Ltd, Niigata, Japan, measuring range: 0 to 100 kg, Grip width range: 20 to 50 mm). The dynamometer was adjusted to sex and the grip span was range 40 to 50 mm. Preschoolers were instructed to gradually and continuously tighten their grip for at least 3-seconds, performing the test twice (alternately with both hands, 10 seconds of rest were allowed between each measurement). However, if the difference among these attempts was >10%, one or two additional attempts were performed until two measurements with less than 10% of variability were obtained. The average of the values for the left and right sides was taken as the final value for handgrip strength to avoid differences between sides in kilogram (kg), without consideration for hand dominance. We periodically calibrated the dynamometer against known weights and found no evidence of drift (2).

Motor component

Speed/agility test (speed of movement, agility and coordination assessment). Two parallel lines were drawn on the ground (with ribbon) 10 meters apart from each other. The preschooler ran as fast as possible from the starting line to the other line and returned to the starting line, crossing each line with both feet every time. The participant had to cross each line with both feet at a time, covering a distance of 40 meters (4x10 meters). Whenever the participant passed a line, he/she would pick up a sponge that had been placed previously. The best of two attempts was recorded (seconds). A slip-proof floor, four cones, a stopwatch and three sponges were used to perform the test (2) (lowest duration in seconds). Before performing this test, a familiarization time (with one attempt) was applied.

Flexibility component

Hamstring and lumbar extensibility were measured using the sit and reach test according in the Australian Council for Health Physical Education and Recreation battery (3). Participants were asked to sit on the floor with legs out straight ahead. The child bent his/her trunk and reached forward as much as possible from the sitting position. Slowly and gradually, he/she pushed a ruler forward with his/her outstretched hand (palm down), without bumps, and stayed in this position for 1-2 seconds. The result was recorded directly from the meter on the device (2,3).

References

1. Cadenas-Sanchez C, Martinez-Tellez B, Sanchez-Delgado G, Mora-Gonzalez J, Castro-Piñero J, Löf M, et al. Assessing physical fitness in preschool children: Feasibility, reliability and practical recommendations for the PREFIT battery. J Sci Med Sport. 2016;19(11):910-5.
2. Ramírez-Vélez R, Rodrigues-Bezerra D, Correa-Bautista JE, Izquierdo M, Lobelo F. Reliability of health-related physical fitness tests among Colombian children and adolescents: the FUPRECOL study. PLoS One 2015;10(10):e0140875.
3. Australian Council for Health Physical Education and Recreation (ACHPER). Handbook for the Australian Fitness Education Award manual (1996) South Australia: ACHPER Publications.

Additional File 2

Table 1. Comparison of the reliability of fitness tests between Fuprecol Kids study and PREFIT study.

**Parameter/tests**	**Mean differences (standard deviation T2-T1)**			
	**Boys**		**Girls**	
	**Fuprecol kids**	**PREFIT Project [1]**	**Fuprecol kids**	**PREFIT Project [1]**
Weight (kg)	–0.01 ± 0.15	–0.01 ± 1.21	–0.01 ± 0.13	0.12 ± 0.90
Height (m)	–0.01 ± 0.01*	0.27 ± 2.09	–0.01 ± 0.04	0.16 ± 1.12
Body mass index (kg/m^2)	0.03 ± 0.23	–0.08 ± 0.68	0.04 ± 0.18	0.11 ± 1.07
Waist circumference (cm)	–0.25 ± 0.23*	–0.19 ± 1.78	0.30 ± 0.98*	0.07 ± 1.60
20 m shuttle run (stage)#	0.25 ± 0.86*	1.02 ± 7.98	0.33 ± 0.87*	3.41 ± 6.87
Handgrip strength (kg)	–0.08 ± 0.58	-0.38 ± 1.32	0.05 ± 0.72	-0.05 ± 1.21
Standing long jump (cm)	–0.56 ± 3.48	–7.51 ± 14.34*	0.35 ± 4.28	–7.03 ± 14.59*
4×10 m shuttle run (s)	0.06 ± 0.96	0.07 ± 0.92	0.42 ± 1.67	0.21 ± 1.15

*In these cases we consider that the reliability of fitness tests markedly differs.
The PREFIT study used laps instead stage, which was used in the present study

Reference

1. Cadenas-Sanchez C, Martinez-Tellez B, Sanchez-Delgado G, Mora-Gonzalez J, Castro-Piñero J, Löf M, et al. Assessing physical fitness in preschool children: Feasibility, reliability and practical recommendations for the PREFIT battery. J Sci Med Sport. 2016;19(11):910-5.
